# Preparation and activity study of Ruoqiang jujube polysaccharide copper chelate

**DOI:** 10.3389/fphar.2023.1347817

**Published:** 2024-01-11

**Authors:** Aierpati Moheteer, Jianlong Li, Xireli Abulikemu, Shakeel Ahmed Lakho, Yan Meng, Jiayi Zhang, Faiz Muhammad Khand, Ambreen Leghari, Saifuding Abula, Qingyong Guo, Dandan Liu, Zhanhai Mai, Waresi Tuersong, Adelijiang Wusiman

**Affiliations:** ^1^ Xinjiang Key Laboratory of New Drug Study and Creation for Herbivorous Animal, College of Veterinary Medicine, Xinjiang Agricultural University, Urumqi, China; ^2^ Laboratory of Traditional Chinese Veterinary Medicine, College of Veterinary Medicine, Xinjiang Agricultural University, Urumqi, China; ^3^ Veterinary and Animal Sciences Sakrand, Shaheed Benazir Bhutto University, Sakrand, Pakistan

**Keywords:** Ruoqiang jujube polysaccharide, polysaccharide metal chelate, copper ion supplementation, immune enhancement, intestinal flora

## Abstract

**Background:** Polysaccharide metal chelate exhibit both immunoregulatory activity and metal element supplementation effects.

**Methods:** In this study, Ruoqiang jujube polysaccharide copper chelate (RJP-Cu) was prepared and the preparation conditions were optimized using the response surface method. Subsequently, RJP-Cu was administered to lambs to evaluate its impact on growth performance, copper ion (Cu^2+^) supplementation, immune enhancement, and intestinal flora was evaluated.

**Results:** The results indicated that optimal RJP-Cu chelation conditions included a sodium citrate content of 0.5 g, a reaction temperature of 50°C, and a solution pH of 8.0, resulting in a Cu^2+^ concentration of 583°mg/kg in RJP-Cu. Scanning electron microscopy (SEM) revealed significant structural changes in RJP before and after chelation. RJP-Cu displaying characteristic peaks of both polysaccharides and Cu^2+^ chelates. Blood routine indexes showed no significant differences among the RJP-Cu-High dose group (RJP-Cu-H), RJP-Cu-Medium dose group (RJP-Cu-M), RJP-Cu-low dose group (RJP-Cu-L) and the control group (*p* > 0.05). However, compared with the control group, the RJP-Cu-H, M, and L dose groups significantly enhanced lamb production performance (*p* < 0.05). Furthermore, RJP-Cu-H, M, and L dose groups significantly increased serum Cu^2+^ concentration, total antioxidant capacity (T-AOC), catalase (CAT), and total superoxide dismutase (T-SOD) contents compared with control group (*p* < 0.05). The RJP-Cu-H group exhibited significant increases in serum IgA and IgG antibodies, as well as the secretion of cytokines IL-2, IL-4, and TNF-α compared to the control group (p < 0.05). Furthermore, RJP-Cu-H group increased the species abundance of lamb intestinal microbiota, abundance and quantity of beneficial bacteria, and decrease the abundance and quantity of harmful bacteria. The RJP-Cu-H led to the promotion of the synthesis of various Short Chain Fatty Acids (SCFAs), improvements in atrazine degradation and clavulanic acid biosynthesis in lambs, while reducing cell apoptosis and lipopolysaccharide biosynthesis.

**Conclusion:** Thus, these findings demonstrate that RJP-Cu, as a metal chelate, could effectively promote lamb growth performance, increase Cu^2+^ content, and potentially induce positive immunomodulatory effects by regulating antioxidant enzymes, antibodies, cytokines, intestinal flora, and related metabolic pathways.

## 1 Introduction

Ruoqiang Jujube Polysaccharide (RJP) is a naturally occurring plant-derived polysaccharide renowned for its diverse nutrient composition. With a complex polysaccharide structure, RJP encompasses various sugar molecules such as glucose and fructose. RJP demonstrates multifaceted benefits, including growth promotion, antioxidant, anti-inflammatory properties, immune cell proliferation, strengthen the immune system, and enhance the body resistance. (Wu Z et al., 2019). The Cu^2+^, an essential trace element, plays a crucial role in numerous cellular mechanisms and signaling pathways within animal organisms (Lelièvre P et al., 2020; Anuk AT et al., 2021; Cheng F et al., 2022). Cu^2+^ deficiency in lambs can lead to various pathological changes such as anemia, nervous dysfunction, movement disorders, coat fading, joint deformation, osteoporosis, decreased vascular wall elasticity, and fertility issues (Helmer C et al., 2021; Yuan S et al., 2022).

Aksu City, situated in the central part of Xinjiang, faces the challenges of a dry and arid climate due to desert airflow, resulting in predominantly sandy loam or sandy saline soil (Zhang et al., 2020). Our previous laboratory investigation revealed that the Cu^2+^ levels in both soil and forage grass in the Aksu region were significantly lower than that of normal soil Cu^2+^ content, emphasizing the need for effective Cu^2+^ supplementation methods (Wumaer et al., 2015). The conventional method for supplementing Cu^2+^ involves using Cu^2+^ sulfate solution. While this method poses risks of liver dysfunction and necrosis due to a swift increase in liver Cu^2+^ (Jin X et al., 2023). Consequently, there is a critical need for a safer, gentler treatment approach with high efficacy in addressing Cu^2+^ deficiency in lambs.

In recent years, plant polysaccharide metal chelates have shown a good application prospect in the livestock industry. As a complex carbohydrate, polysaccharide has the ability to chelate metal ions, thereby improving the bioavailability of metal ions and helping to maintain the normal physiological function of the body. Notably, polysaccharide-Cu^2+^ chelates have demonstrated the potential to improve animal growth performance and exhibit significant intestinal immune-boosting functions (Zhang J et al., 2023). Various studies have highlighted the antioxidant and multifunctional iron supplement potential of polysaccharide metal chelates, including dioscorea polysaccharide iron chelate (DTP-Fe), which can enhance the scavenging of DPPH free radicals (Liu Y et al., 2023), ABTS+ and hydroxyl free radicals in the organism, with the antioxidant capacity of TDP-Fe increasing with higher concentrations. Moreover, research showed that chelation of Enteromorpha polysaccharide with iron (III) or zinc (II) can alter the structure of Enteromorpha polysaccharide and enhance its anti-inflammatory activity (Feng Y et al., 2023). Chelation with different metal ions, such as Fe, Zn, Mg, Cr, and Pt, has been reported to enhance the biological activity of polysaccharides (Li X et al., 2022). It has been reported that chelating Fe (III), Zn (II), and Cr (III) with corn beard polysaccharide could alter the morphology, conformation, thermal stability, and biological activity of corn beard polysaccharide (Jia Y et al., 2021). Zinc chelates of corn palp polysaccharide exhibited higher antioxidant activity and a more pronounced inhibitory effect on α-glucosidase than corn palp polysaccharide.

This experiment aims to chemically chelate RJP with CuSO_4_ to prepare an RJP-Cu with copper supplementation and immune-enhancing activity. Therefore, optimization of the experimental conditions for RJP-Cu was conducted and is impact on lamb growth performance and immune-enhancing activity was investigated. To further explores the regulatory effects of RJP-Cu on lamb intestinal microbiota and Short-Chain Fatty Acids (SCFAs) through 16S rDNA sequencing and gas chromatography, respectively.

## 2 Materials and methods

### 2.1 Animal experiments

The experimental subjects comprised of 40 lambs, approximately 1 month old, with an average weight of 6.30 ± 0.10 kg. All animal experiments were carried out at the Baodi farm in Kalatale Town, Aksu City. The Experimental Animal Welfare Ethics Committee of Xinjiang Agricultural University approved the experiment (approval number: 2022016) to ensure compliance with all relevant ethical regulations for animal experiments and research.

### 2.2 Main reagents and instruments

The Glutathione assay kit, Malondialdehyde (MDA) assay kit, Catalase assay kit, Total Superoxide Dismutase assay kit, and Total Antioxidant Capacity (T-AOC) assay kit were all purchased from Nanjing Jiengcheng Biotechnology Co., Ltd. The Interleukin-2 (IL-2) test kit, Interleukin-4 (IL-4) test kit, Tumor Necrosis Factor-α (TNF-α) test kit, Immunoglobulin G (IgG) test kit, Immunoglobulin A (IgA) test kit, and Cu^2+^ colorimetric reagent is purchased from Shanghai Vanco Bio-Technology Co., Ltd. Other equipment used in the study included a Bio Tek Instruments (Icn), a Veterinary Blood Analyzer (ABAXIS Europe), a Centrifuge (TDL-40B) from Shanghai Anting Scientific Instrument Factory, and a Flame Atomic Absorption Spectrophotometer (SP-3520 AA) from Shanghai Yili Scientific Instrument Co., Ltd.

### 2.3 Extraction of RJP and preparation of RJP-Cu

The RJP extraction involved weighing and immersing Ruoqiang jujube in anhydrous ethanol and petroleum ether for degreasing and drying. The dried sample was boiled and extracted, and the resulting solution is concentrated to a concentration of 1 g/mL. The concentrated liquid is then mixed with Sevag reagent (chloroform: n-butanol = 4:1) at a volume ratio of V_RJP_: V_Sevag reagent_ = 2:5. The mixture was vigorously shaken, left to stand for 30 min, and then centrifuged for 10 min at 4000 rpm/min. The aqueous phase was collected through three repetitions. Anhydrous ethanol is then added to achieve an 80% ethanol concentration. The mixture was refrigerated at 4°C overnight, and the precipitate, washed and dried with anhydrous ethanol to obtain dry RJP material.

**FIGURE 1:**
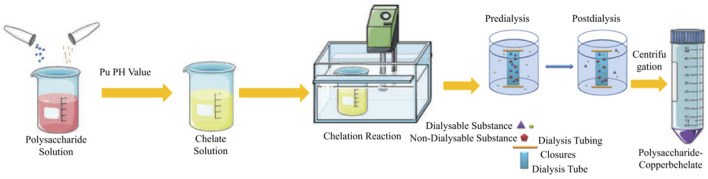
Preparation diagram of RJP-Cu.

The preparation of RJP-Cu involved combining extracted RJP and sodium citrate in 300 mL of water. The mixture was heated in a water bath, stirred continuously and pH adjusted using a 20% NaOH solution. CuSO_4_ was added slowly, stirring ceased when insoluble matter appeared, and the mixture was centrifuged at 4000 rpm for 1 min to obtain the supernatant. The liquid was collected, concentrated, cooled, and anhydrous ethanol was added. After refrigerating at 4°C overnight, the mixture was centrifuged at 4000 rpm for 1 min to precipitate. RJP-Cu was dissolved in water at a ratio of 1:10. A dialysis bag, soaked in flowing water for 24 h, concentrated the remaining liquid. After alcohol precipitation, drying, and refinement, RJP-Cu was obtained (Figure 1).

### 2.4 One-factor experimental design of RJP-Cu

To assess the influence of sodium citrate content on Cu^2+^ concentration, the reaction time was set at 1 h, temperature at 60°C, pH at 9.0, and sodium citrate content at 0.0625 g, 0.125 g, 0.25 g, 0.5 g, and 1 g, respectively. To assess the influence of reaction temperature on Cu^2+^ concentration.

To assess the influence of reaction temperature on Cu^2+^ concentration, the reaction time fixed at 1 h, pH at 9.0, and sodium citrate content at 0.5 g, the reaction temperature was varied at 30°C, 40°C, 50°C, 60°C, and 70°C. To assess the influence of pH value on Cu^2+^ concentration, the reaction time was set at 1 h, temperature at 50°C, and sodium citrate content at 0.5 g, the pH value was adjusted to 7.0, 8.0, 9.0, 10, and 11, respectively. In each of the aforementioned experimental groups, the average value was determined based on three repetitions, and a significance analysis was conducted to identify the impact of different single factors on the RJP-Cu.

### 2.5 Response surface experimental design

According to the results of univariate experimental, sodium citrate content (A), reaction temperature (B) and pH value (C) were selected as key variables. −1, 0 and +1 indicated low, medium and high levels respectively, and the response surface analysis of 3 factors and 3 levels was conducted. The design factor level is shown in (Table 1).

**TABLE 1:** Experimental factors and levels of the response surface.

Level	Factor		
	A: sodium citrate content (g)	B: reaction temperature (°C)	C: PH value
−1	0.25	40	7
0	0.5	50	8
1	1	60	9

### 2.6 Preparation conditions of RJP-Cu and preparation of RJP-Cu

Based on the response surface experimental results, RJP-Cu was prepared under the following conditions: a reaction time of 1 h, a sodium citrate content of 2.5 g, a reaction temperature of 50°C, and a pH value of 8.0. RJP-Cu was completely dissolved in distilled water, and a 1000 Da molecular weight cutoff dialysis bag was employed for 2 days to concentrate the dialyzed RJP-Cu. RJP-Cu, with free impurities removed, was obtained after alcohol precipitation.

### 2.7 Determination of RJP and RJP-Cu content

The polysaccharide content in RJP and RJP-Cu was determined using the phenol-sulfuric acid method (Yue F et al., 2022). The protein content in RJP and RJP-Cu was assessed using the BCA kit method (Rogatsky E, 2021). The glucuronic acid content in RJP and RJP-Cu was measured using the sulfuric acid-carbazole method (Maccari F and Volpi N, 2022). The Cu^2+^ in the samples was analyzed via flame atomic absorption spectrometry (Bakircioglu D et al., 2022).

### 2.8 Scanning electron microscopy and infrared spectroscopy

RJP and RJP-Cu samples were prepared for SEM analysis to observe the shape and characteristics of the samples (Mobin M and Rizvi M, 2017). The infrared spectra (FT-IR) of RJP and RJP-Cu were measured using a Fourier transform infrared spectrometer. A small amount of each sample mixed with KBr particles, was ground into a powder, dried, and then pressed into thin slices. These slices were subsequently scanned and measured in the range of 400–4000 cm⁻^1^ (Yi Y et al., 2020).

### 2.9 Animal classification and administration of medication

**FIGURE 2:**
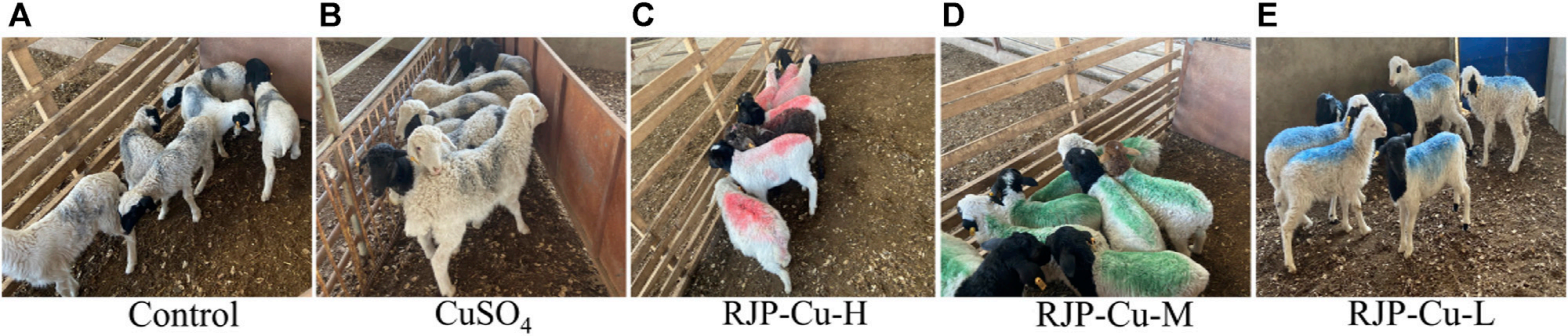
Group of lambs. **(A)** Control. **(B)** CuSO4. **(C)** RJP-Cu-H. **(D)** RJP-Cu-M. **(E)** RJP-Cu-L.

Forty lambs with similar organism weights were selected. After 14 days of adaptation to the feeding regimen, the lambs were divided in to 5 groups based on their organism weights, with 8 lambs in each group. These groups were as follows: the Cu sulfate group (CuSO_4_, 4 mg/kg), the RJP-Cu high-dose group (RJP-Cu-H, 5.3 mg/kg), the RJP-Cu medium-dose group (RJP-Cu-M, 2.6 mg/kg), the RJP-Cu low-dose group (RJP-Cu-L, 1.3 mg/kg), and the blank control group, blank control group received no additional treatment (Figure 2).

After a total of 14 days of feeding, all groups were maintained on a basal diet for an additional 28 days. The experimental lambs were fed in groups and had free access to water. Feeding occurred once daily at 10:00 and 19:00, each feeding session lasting for 1.5 h. Any remaining feed was collected at 11:30 and 20:30 daily. The feeding mode, management practices, and environmental conditions were consistent for all groups throughout the duration of the experiment.

### 2.10 Effect of RJP-Cu on growth performance of lambs

The feeding conditions for each group of lambs were meticulously recorded on a daily basis throughout the entire experiment. Additionally, the organism mass of each lamb was weighed once a week. Measurements for organism height, organism diagonal length, chest circumference, and tube circumference were taken at the beginning and end of the experiment. Various metrics such as average daily gain, average daily feed intake, feed-to-weight gain ratio, organism height, organism diagonal length, chest circumference, and tube circumference were calculated. Furthermore, blood samples were collected from the jugular vein of the lambs on the 28th day after feeding.

### 2.11 Effects of RJP-Cu on serum copper content, oxidation indexes, immunoglobulin and cytokine contents of lambs

On the 28th day following the cessation of feeding, blood samples were gathered for the assessment of T-AOC, CAT, GSH-PX, MDA, and SOD. Additionally, on the 14th and 28th days after the termination of feeding, blood samples were obtained to measure the Cu^2+^ in the serum using a Cu^2+^ analysis kit and the levels of IgG, IgA, IL-2, IL-4, TNF-α were measured using a ELISA kits.

### 2.12 Effect of RJP-Cu on the intestinal microbiota in lambs

On the 28th day following the cessation of feeding all groups of lambs were administered a 15 mL normal saline rectal injection, and rectal feces were collected. The collected samples were preserved in liquid nitrogen tanks and transported to the laboratory for DNA extraction. DNA quantification was carried out using nanodrop, and following purification, PCR amplification and fluorescence quantification were performed. Subsequently, a sequencing library was prepared for computer-based sequencing. Nomi Biological Company conducted the analysis of intestinal microorganisms and indexes related to SCFAs.

### 2.13 Statistical analysis

SPSS 24.0 software is suitable for conducting single-factor experimental analysis to determine significant differences. Design Expert 10.0.7 software is employed for the statistical analysis of response surface experimental outcomes.

## 3 Results and analysis

### 3.1 Single factor test results

#### 3.1.1 Effect of sodium citrate content, reaction temperature, and pH on copper concentration in RJP-Cu

**FIGURE 3:**
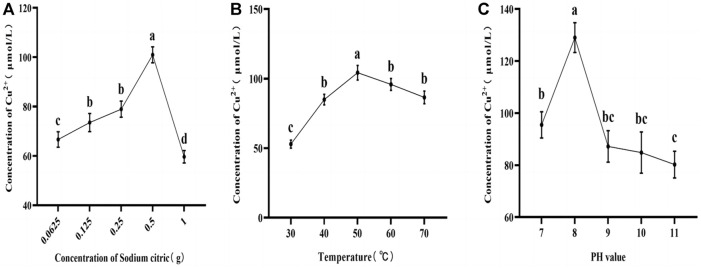
**(A)** Represents the impact of sodium citrate content on Cu^2+^ concentration. **(B)** Depicts the influence of reaction temperature on Cu^2+^ concentration. **(C)** Illustrates the influence of pH value on Cu^2+^ concentration. Note: a, b, c, d letters indicate a significant difference (*p* < 0.05).

As depicted in Figure 3, the influence of sodium citrate content, reaction time, and pH value on the concentration of Cu^2+^ in RJP-Cu exhibited a common pattern of initially increasing and subsequently decreasing. With a constant reaction time of 1 h, a temperature of 60°C, and a pH of 9.0, the Cu^2+^ concentration in RJP-Cu rose with an increase in sodium citrate content, reaching its peak at 0.5 g and subsequently declining rapidly, Cu^2+^ is 100.973 μmol/L (Figure 3A). Under conditions of a fixed extraction time of 1 h, a sodium citrate content of 0.5 g, and a pH of 9.0, Cu^2+^ is 104.265 μmol/L, the concentration of Cu^2+^ in RJP-Cu increased with rising reaction temperature, reaching its maximum at 50°C before gradually decreasing (Figure 3B). Maintaining a consistent reaction time of 1 h, a temperature of 50°C, and a sodium citrate content of 0.5 g, the concentration of Cu^2+^ in RJP-Cu augmented with an increase in pH value, reaching its peak at pH-8, and subsequently declining rapidly, Cu^2+^ is 129.025 μmol/L (Figure 3C).

#### 3.1.2 Response surface experimental results

Design Expert 8.0.6 software was used to optimize the response surface for the factors affecting Cu^2+^ concentration in RJP-Cu: sodium citrate content (A), reaction temperature (B) and pH value (C) were selected as key variables. By comparing the predicted and measured values under the group sum and the last 17 conditions, the regression equation was obtained as follows: Y = −2090.54 + 474.07A + 27.79B + 341.65C-0.84AB-8.29AC-0.34BC-302.41A^2^-0.23B^2^-19.88C^2^ (Table 2).

**TABLE 2:** Response surface experimental protocol and results.

Test number	Factor			Cu^2+^ μmol/L	
	A	B	C	Measured value	Predicted value
1	0.25	50	7	72.832	71.578
2	0.25	50	9	80.231	79.479
3	1	60	8	64.001	64.873
4	0.5	50	8	132.423	130.076
5	0.5	40	9	82.101	84.725
6	0.25	60	8	75.875	80.627
7	1	50	7	67.506	68.382
8	0.5	50	8	125.418	130.076
9	0.5	50	8	130.235	130.076
10	1	40	8	62.256	59.378
11	0.5	60	7	97.542	94.917
12	0.25	40	8	65.203	62.455
13	1	50	9	62.716	63.844
14	0.5	50	8	132.387	130.076
15	0.5	50	8	129.918	130.076

A regression model was analyzed, and the results show that the model had a highly significant statistical significance with a *p*-value of less than 0.0001. The correlation coefficient *R* ^2^ = 0.9913, and the Adjusted *R* ^2^ = 0.9802, both of which are close to 1.000. Moreover, the coefficient of variation CV = 4.31%, indicating a strong fit of the model and suitability for the actual extraction process. The optimal extraction process for RJP-Cu was determined as follows: a sodium citrate content of 0.5 g, a reaction temperature of 50°C, and a pH value of 8. The predicted Cu^2+^ concentration was 130.067 μmol/L, while the actual measured Cu^2+^ concentration was 132.423 μmol/L (Table 3).

**TABLE 3:** Results of the regression model ANOVA.

	Quadratic sum	Free party	Mean square	F	P	Significance
Model	12343.67	9	1371.52	89.10	<0.0001	significant
A-A	177.30	1	177.30	11.52	0.0115	
B-B	266.09	1	266.09	17.29	0.0043	
C-C	5.37	1	5.37	0.3489	0.5733	
AB	42.41	1	42.41	2.76	0.1409	
AC	40.84	1	40.84	2.65	0.1474	
BC	48.99	1	48.99	3.18	0.1176	
A^2^	5684.45	1	5684.45	369.30	<0.0001	
B^2^	2399.26	1	2399.26	155.87	<0.0001	
C^2^	1664.64	1	1664.64	108.15	<0.0001	
Residual	107.75	7	15.39			
Lack of Fit	75.15	3	25.05	3.07	0.1532	not significant
Pure Error	32.60	4	8.15			
Cor Total	12451.42	16				

#### 3.1.3 Results of the RJP and RJP-Cu content assays

Standard curves were established to derive regression equations: Y = 7.3275 X+0.0999 (*R* ^2^ = 0.9976) for polysaccharides, Y = 3.455 X+0.1263 (*R* ^2^ = 0.9978) for proteins, and Y = 2.8529 X+0.0471 (*R* ^2^ = 0.9995) for uronic acid. The total polysaccharide, protein, and uronic acid contents in RJP and RJP-Cu were determined using these regression equations, while Cu^2+^ was measured by flame atomic absorption spectrometry.

As indicated in Table 4, there were no significant differences in total polysaccharide and protein contents between RJP and RJP-Cu (*p* > 0.05). However, the uronic acid content in RJP was significantly reduced compared to RJP-Cu (*p* < 0.05), while the Cu^2+^ concentration significantly increased to 134.423 μmol/L.

**TABLE 4:** Percentage of different index content between RJP and RJP-Cu.

Indicator	Total polysaccharides	Protein	Uronic acid	Cu^2+^
RJP	72.4 ± 0.12%^a^	2.8 ± 0.1%^a^	14.2 ± 0.13%^a^	0.4 μmol/L/g^b^
RJP-Cu	73.5 ± 0.16%^a^	2.5 ± 0.18%^a^	5.0 ± 0.11%^b^	132.423 µmol/L^a^

Note: a, b letters indicate a significant difference (*p* < 0.05).

#### 3.2 Morphology of RJP and RJP-Cu by scanning electron microscopy

**FIGURE 4:**
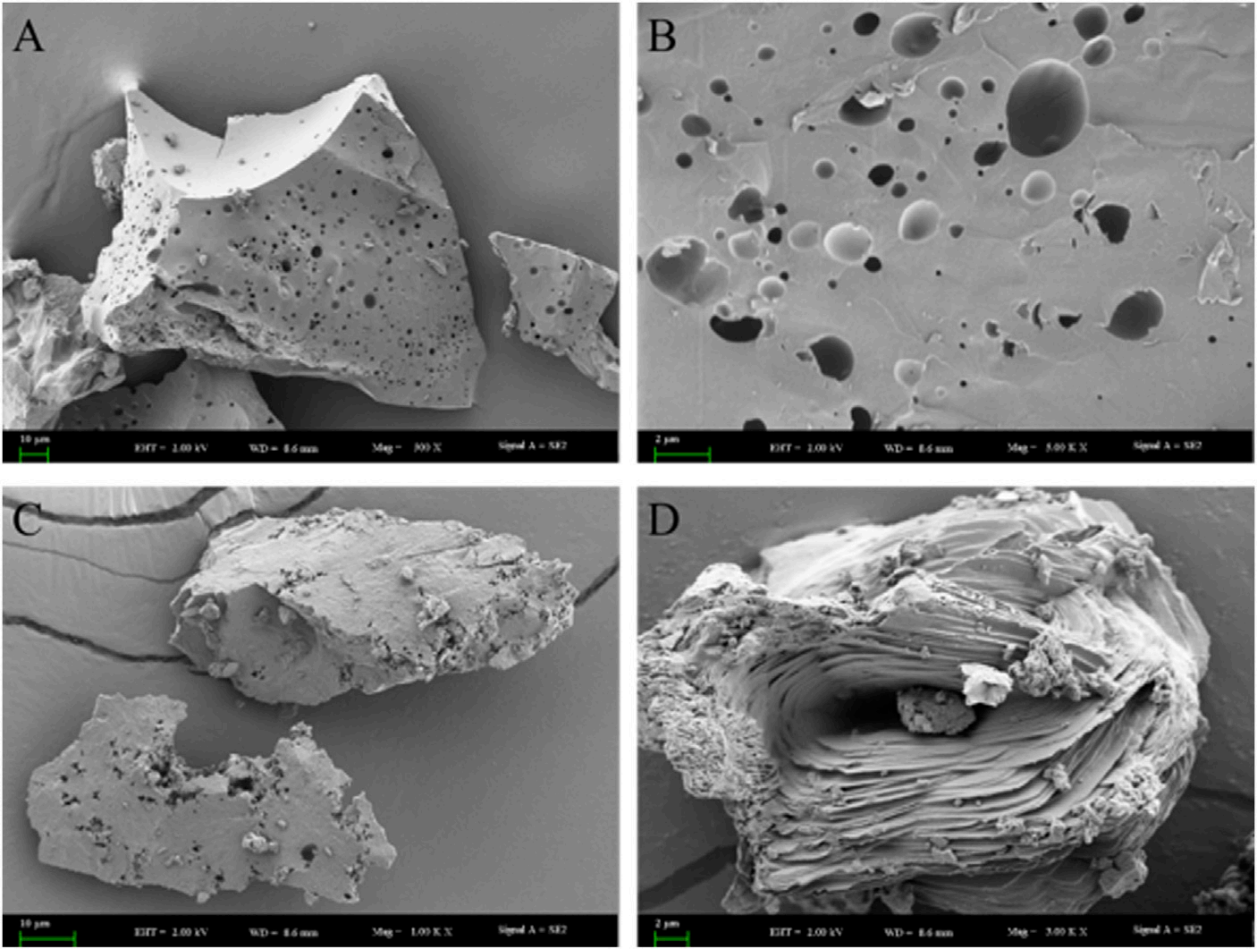
**(A,B)** Correspond to scanning electron microscope images of RJP. **(C,D)**: Correspond to scanning electron microscope images of RJP-Cu.

As shown in Figure 4A, the SEM morphology of RJP revealed a smooth, porous surface and a dense texture structure. The surface of RJP appeared uneven, featuring various hole sizes and depths (Figure 4B). In contrast, the SEM morphology of RJP-Cu exhibited a more open and loose structure with rough edges (Figure 4C). It displayed an irregular structure with visible cracks and holes on the surface, suggesting that RJP-Cu is composed of stacked lamellar structures with rough edges (Figure 4D). It is evident that the electron microscope structures of RJP and RJP-Cu differ obviously.

### 3.3 RJP and RJP-Cu infrared spectroscopy of analysis

**FIGURE 5:**
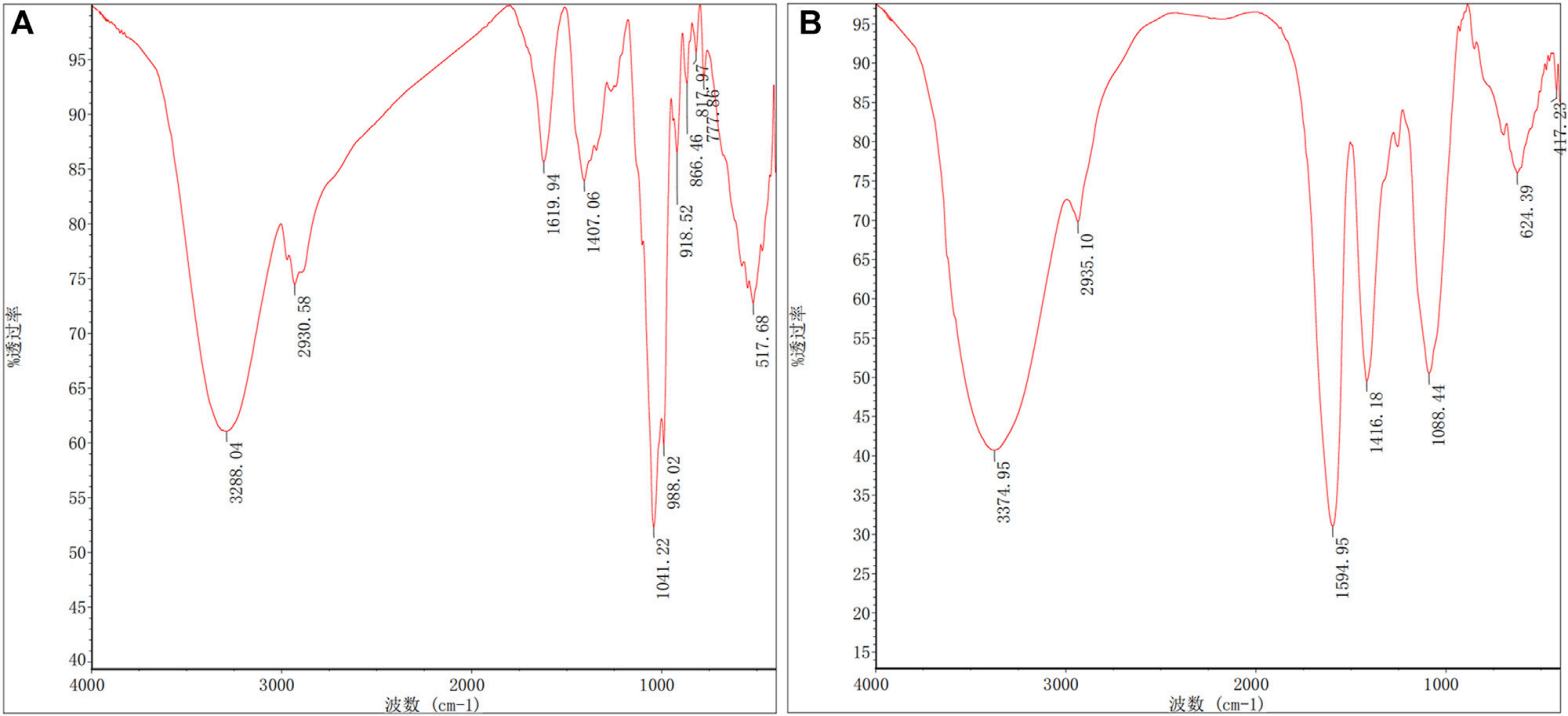
Infrared spectra of **(A)** RJP and **(B)** RJP-Cu.

RJP and RJP-Cu exhibited prominent and extensive hydroxyl (-OH) stretching vibration absorption peaks, along with moderately strong (-CH_2_-) C-H bond stretching vibration peaks in the range of 3400–3200 cm^-1^ and 3000–2800 cm^-1^, respectively. These peaks are characteristic of polysaccharide absorption. The similarity in their position, shape, and intensity indicates a shared carbon chain structure and monosaccharide composition. Upon Cu^2+^ chelation, RJP displayed a stretching vibration peak of -CH_2_OH (3288.04–2930.58 cm^-1^), while the C=O stretching vibration peak at 1619.94 cm^-1^ disappeared. Furthermore, changes in C-O stretching (1407.06 cm^-1^, 1041.22 cm^-1^) and a shift in the C-O wave number (from 1407.06 cm^-1^ to 1416.18 cm^-1^) suggest a strong binding affinity of Cu^2+^ to the O in C-O, indicating a chemical interaction. The characteristic absorption peak at 1594.95 cm^-1^ in RJP-Cu indicates the presence of SO_4_^2-^, implying that RJP simultaneously chelates SO_4_^2-^ along with Cu^2+^, a form of physical chelation. These results affirm that Cu^2+^ forms both chemical and physical chelation bonds with RJP, resulting in the formation of RJP-Cu (Figure 5).

### 3.4 Effects of RJP-Cu on lamb blood routine indexes

As indicated in Table 4, compared to the control group, there were no statistically significant differences in the counts of leukocytes, lymphocytes, monocytes, neutrophil granulocytes, red blood cells, hematocrit levels, or platelets between the RJP-Cu-H, M, and L groups (*p* > 0.05). This suggests a high level of safety. This indicates that all doses of RJP-Cu have a good safety profile.

### 3.5 Effects of RJP-Cu on daily weight gain and feed intake in lambs

The final weight, daily weight gain, and daily feed intake of lambs in the RJP-Cu-H, M, and L groups were significantly higher than the control group (*p* < 0.05), and all indicators were measured exhibiting a dose-dependent pattern. Furthermore, the feed-to-gain ratio in the RJP-Cu-H, M, and L groups was significantly lower than that in the control group (*p* < 0.05). This suggests that RJP-Cu effectively promotes lamb growth and improves feed utilization efficiency (Table 6).

### 3.6 Effect of RJP-Cu on organism ruler indexes in lambs

There were no significant differences in the initial organism height, initial oblique organism length, initial chest circumference, and initial tube circumference were observed among all experimental groups (*p* > 0.05). However, the final organism height, final oblique organism length, final chest circumference, and final tube circumference in the RJP-Cu-H, M, and L groups were significantly higher than those in the control group (*p* < 0.05), and this increase was dose-dependent. These results suggest that RJP-Cu effectively promotes the growth and bone development of lambs (Table 7).

### 3.7 Effect of RJP-Cu on serum copper content in lambs

**FIGURE 6:**
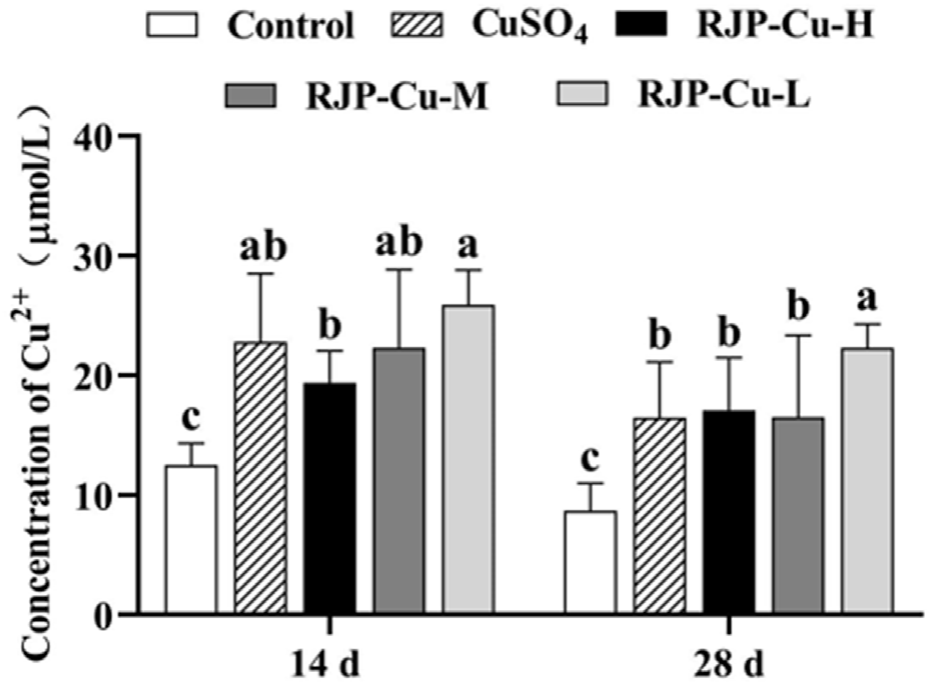
The Impact of RJP-Cu on Serum Cu in Lambs. Note: a, b, c letters indicate a significant difference (*p* < 0.05).

The serum Cu^2^⁺ levels in the CuSO_4_ group, as well as the RJP-Cu-H, M, and L groups, were significantly higher than those in the control group on both day 14 and day 28 (*p* < 0.05). This indicates that the CuSO_4_ group and the RJP-Cu-H, M, and L groups all demonstrated a notable and effective Cu^2+^ supplementation effect (Figure 6).

### 3.8 Effect of RJP-Cu on oxidative serum indexes in lambs

As indicated in Table 8, the T-AOC, CAT, and T-SOD levels in the RJP-Cu-H, M, and L groups were significantly higher compared to those in the control group (*p* < 0.05). This result suggests that RJP-Cu could effectively eliminate excess free radicals within the organism.

### 3.9 Effect of RJP-Cu on serum immunoglobulin content in lambs

**FIGURE 7:**
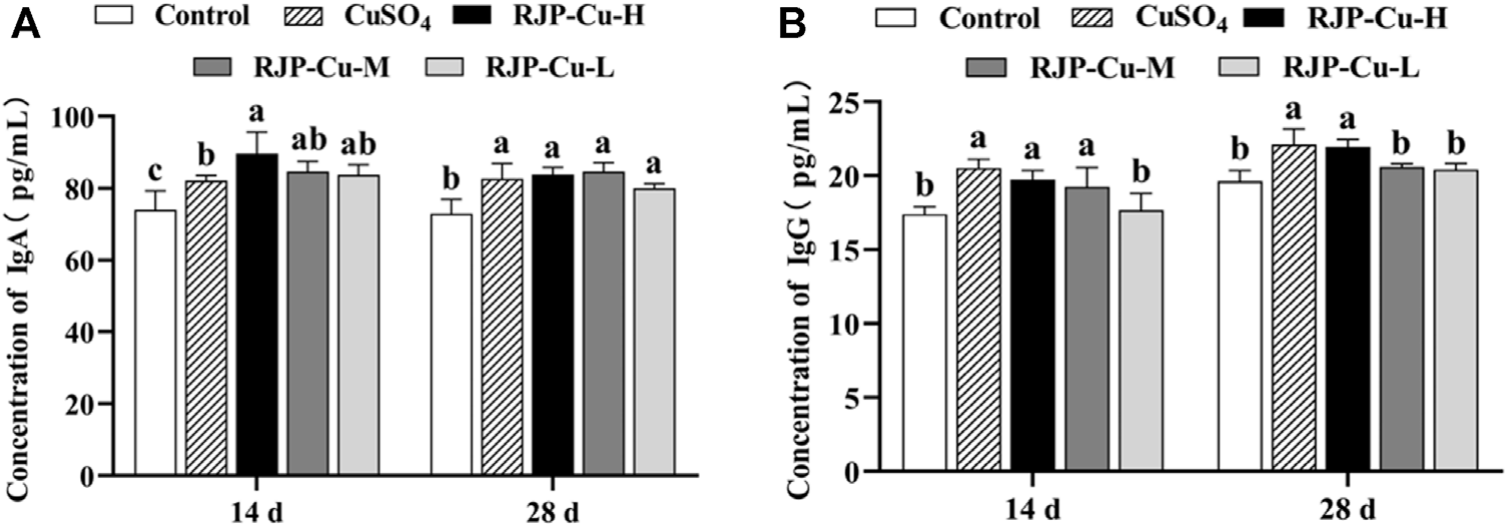
The Influence of RJP-Cu on Serum Immunoglobulin Content in Lambs. **(A)** Concentration of IgA. **(B)** Concentration of IgG. Note: a, b, c, letters indicate a significant difference (*p* < 0.05).

The serum IgA levels in lambs from the RJP-Cu-H, M, L groups, and the CuSO_4_ group were significantly higher than those in the control group on both day 14 and day 28 (*p* < 0.05). Specifically, on day 14, the serum IgA levels in lambs from the RJP-Cu-H group were significantly higher than those in the CuSO_4_ group (*p* < 0.05) (Figure 7A). Furthermore, it is evident that the serum IgG levels in lambs from the RJP-Cu-H, M, and CuSO_4_ groups on day 14 were significantly higher compared to the control group (*p* < 0.05). On day 28, the serum IgG levels in lambs from the RJP-Cu-H group and the CuSO_4_ group were significantly higher than those in the control group (*p* < 0.05). These results indicate that the RJP-Cu-H group effectively stimulates the production of IgA and IgG in lamb serum (Figure 7B).

### 3.10 Effect of RJP-Cu on serum cytokine content in lambs

**FIGURE 8:**
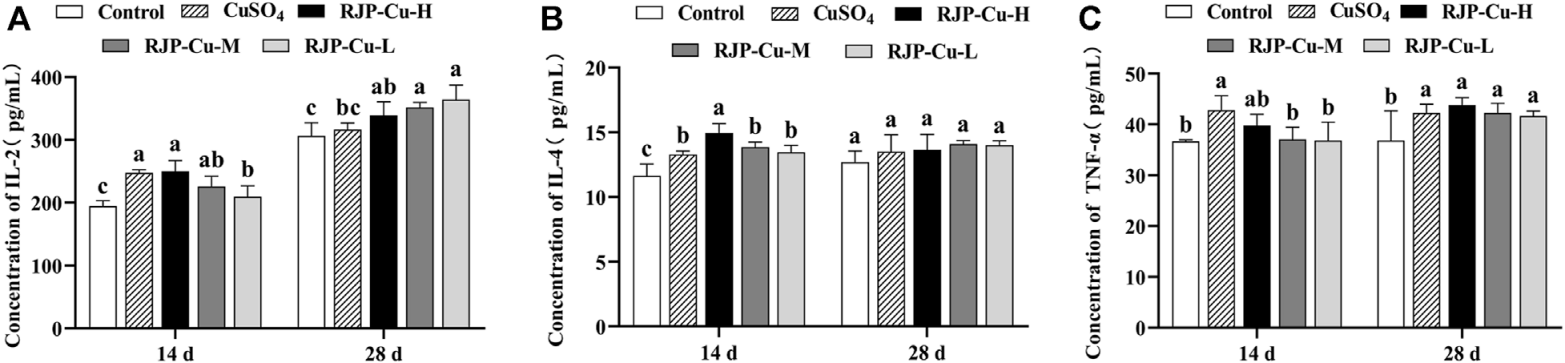
The Impact of RJP-Cu on Serum Cytokine Content in Lambs. **(A)** Concentration of IL-2. **(B)** Concentration of IL-4. **(C)** Concentration of TNF-α. Note: a, b, c, letters indicate a significant difference (*p* < 0.05).

The serum interleukin-2 (IL-2) levels in the RJP-Cu-H group and CuSO_4_ group were significantly higher than those in the RJP-C-L group and control group on day 14(*p* < 0.05). On day 28, the serum IL-2 levels in the RJP-Cu-H, M, and L groups were significantly higher than those in the control group (*p* < 0.05) (Figure 8A). Additionally, IL-4 content in the RJP-Cu-H group was significantly higher than that in the RJP-Cu-M, L, CuSO_4_, and control groups on day 14 (*p* < 0.05). On day 28, no significant differences were observed in IL-4 content among all groups (*p* > 0.05) (Figure 8B). TNF-α content in the CuSO_4_ group was significantly higher than that in the RJP-Cu-M and L, and control groups on day 14 (*p* < 0.05), with no significant difference observed between the RJP-Cu-H group and the CuSO_4_ group (*p* > 0.05). On day 28, the TNF-α content in all experimental groups was significantly higher than that in the control group (*p* < 0.05) (Figure 8C). These results indicate that the RJP-Cu-H group can enhance the secretion of IL-2, IL-4, and TNF-α in the serum of lambs.

### 3.11 Effects of RJP-Cu on intestinal flora diversity and richness in lambs

**FIGURE 9:**
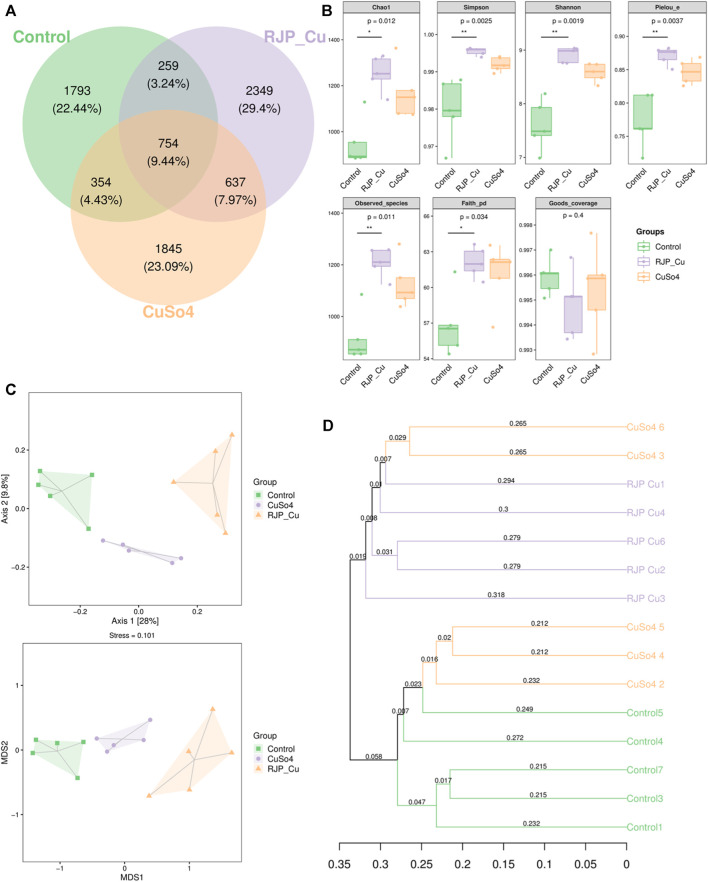
α-diversity and β-diversity analysis of intestinal flora in lambs. **(A)** Venn diagram based on OTU level. **(B)** β-Diversity analysis of the intestinal flora. **(C)** Principal Coordinate Analysis (PCoA) of bacterial communities using Jaccard distance. **(D)** UPGMA clustering tree based on sample distance matrix.

The number of unique Operational Taxonomic Units (OTUs) for the control, RJP-Cu, and CuSO_4_ groups were 1793, 2349, and 1845, respectively. There were 754 common intestinal flora species shared among the three groups, and 259 OTUs were common between the control and RJP-Cu groups, and 637 OTUs were common between the RJP-Cu and CuSO_4_ groups. In summary, the RJP-Cu-H group exhibited an increased diversity of intestinal microflora species in lambs (Figure 9A). As shown in Figure 9B, the coverage for each group is greater than 0.995 and there is no significant difference among each groups (*p* > 0.05), Chao1, Pielou_e, Observed_species, Faith_pd, Simpson index and Shannon index in RJP-Cu group are significantly higher than those in the control group (*p* < 0.05). Both Principal Coordinate Analysis (PCoA) and Non-Metric Multidimensional Scaling (NMDS) maps revealed differences in the composition of flora among the control, RJP-Cu, and CuSO_4_ groups (Figure 9C). The UPGMA cluster analysis diagram further illustrated that the control group exhibited distinct clustering, and there were noticeable differences in the composition of flora among the control, RJP-Cu, and CuSO_4_ groups (Figure 9D).

### 3.12 Effect of RJP-Cu on intestinal flora composition and structure in lambs

**FIGURE 10:**
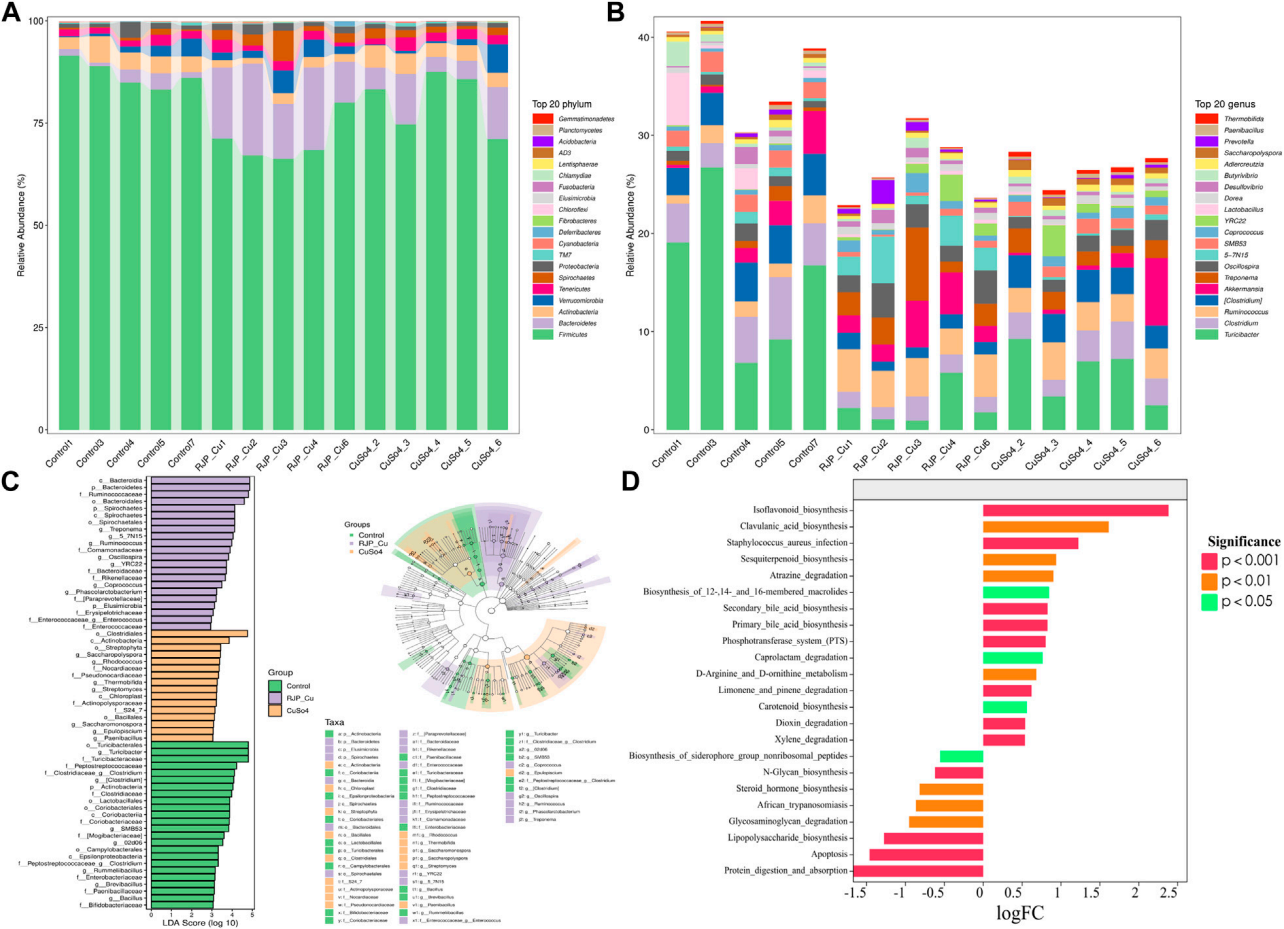
Gate and genus level, Marker species and Functional potential prediction of intestinal flora in lambs. **(A)** Analysis of species composition at the phylum level. **(B)** Analysis of species composition at the Genus level **(C)** Difference analysis method LDA Effect Size (LEfSe). **(D)** Metabolic Pathway Statistics of KEGG.

The RJP-Cu group exhibited an increased abundance of *Spirochaetes*, *Bacteroidetes*, and *Verrucomicrobia* compared to the control group, and the abundance of beneficial bacteria *Actinobacteria* decreased. These results indicated that at phylum level RJP-Cu effectively enhanced the prevalence and quantity of beneficial bacteria and reducing the presence of harmful bacteria, contributing to a healthier composition of the intestinal flora (Figure 10A). At genus level the RJP-Cu group increased the abundance of beneficial bacteria *Prevotella*, *Oscillospira*, *Ruminococcus*, *Coprococcus*, and *Akkermansia*, and decreasing the abundance of the harmful bacteria *Clostridium* compare to the control and CuSO_4_ groups (Figure 10B).

After confirming the regulatory impact of RJP-Cu on bacterial composition at both the phylum and genus levels, LDA EFfect Size (LEfSe) was used to further discern the specific effects of RJP-Cu on lamb intestinal bacteria. Substantial changes were observed in the predominant intestinal flora of the RJP-Cu, CuSO_4_, and control group. Most of the beneficial bacteria in the RJP-Cu group have the potential to promote host intestinal health, while the control group retained a certain amounted of pathogenic bacteria. These findings highlight that RJP-Cu can increase the abundance of beneficial bacteria and reducing the prevalence of harmful bacteria, thereby promoting intestinal health (Figure 10C).

According to the log fold change (logFc) analysis result, compared to the control group, the RJP-Cu group demonstrated a significant increase in processes related to flavonoid biosynthesis (*p* < 0.05), clavulanic acid biosynthesis (*p* < 0.05), siterpenoid synthesis, and atrazine degradation (*p* < 0.05). Conversely, it showed a decrease in activities associated with cell apoptosis and lipopolysaccharide biosynthesis (Figure 10D).

### 3.13 Effect of RJP-Cu on SCFAs in lambs

SCFAs are vital for maintaining intestinal health. The isovaleric acid content in the RJP-Cu group was significantly higher compared to both the CuSO_4_ and control groups (*p* < 0.05). Additionally, the levels of valerate, propionic acid, and isobutyric acid in the RJP-Cu-H group and the CuSO_4_ group were significantly higher than those in the control group (*p* < 0.05). Moreover, the contents of valerate and isobutyric acid in the RJP-Cu group were higher than those in the CuSO_4_ group, but not statistically significant (*p* > 0.05) (Table 9). These findings demonstrate that RJP-Cu can stimulate the synthesis of multiple SCFAs.

## 4 Discussion

In recent years, increasing numbers of studies have shown that polysaccharide metal complexes display diverse biological activities, such as improving growth, antioxidation, immune regulation, and antibacterial activity. Zhang et al. (Zhang J et al., 2023) reported that the supplementation of dietary Chinese yam polysaccharide-Cu complex could improve the indexes of the growth, carcass, serum biochemistry, immunity and oxidation resistance in broilers, such as average daily gain, the slaughter percentage, breast muscle percentage, leg muscle percentage, growth-related hormones insulin-like growth factor I, triiodothyronine, thyroxine, growth hormone, insulin, antibodies and the cytokines, IgM, IgG, IgA, IL-2, IL-4, IL-6, T-SOD, T-AOC, GSH-Px, and GSH-ST. Research showed that soybean polysaccharide iron complex can promote the growth of the beneficial bacteria *Bacillus licheniformisand* inhibit the proliferation of the pathogenic bacteria *Staphylococcus aureus*, thus improving the growth and immunity of the body (Gao W et al., 2020). Due to their safety, growth-promoting effect, and immune-enhancing properties are used as functional feed additives in animal production.

Response surface methodology (RSM) is a powerful statistical technique used to optimize processing parameters, commonly applied in drug extraction, synthesis, and other process variables, as well as the interactions between these variables (Li WF et al., 2021). In this experiment, Design Expert 8.0.6 software was utilized to analyze and optimize the preparation conditions of RJP-Cu. The response surface test for RJP-Cu showed that the influence factors on the Cu^2+^ concentration could be expressed by the second-order polynomial equation, Y = −2090.54 + 474.07A + 27.79B + 341.65C-0.84AB-8.29AC-0.34BC-302.41A^2^-0.23B^2^-19.88C^2^ (Table 3). The ANOVA study for the regression equation results show that the regression model is highly significant (*p* < 0.0001), with a correlation coefficient of *R* ^2^ = 0.9913, AdjR^2^ = 0.9802, all approaching 1.000, and a coefficient of variation of CV = 4.31%, signifying that the model fits well. Under the optimum preparation conditions, the concentration of Cu^2+^ in RJP-Cu is 132.423 μmol/L. Scanning electron microscopy (SEM) and infrared spectroscopy (FT-IR) were used to detect the changes of surface morphology and functional groups of polysaccharides after modification (Gritsch L et al., 2018). As shown in Figure 4, the structure of RJP is greatly changed after chelating with and CuSO_4_. FT-IR detection results showed that RJP-Cu has both characteristic polysaccharide functional groups and Cu^2+^ groups (Figure 5), indicating that Cu^2+^ chemically and physically chelates with RJP.

Blood routine analysis is a critical indicator of overall health (Wang J et al., 2022). As shown in Table 5, there were no significant differences in white blood cell count, lymphocytes, red blood cells, hemoglobin, and platelets between the RJP-Cu group and the control group (*p* > 0.05), indicating that RJP-Cu is safe for consumption. Lamb growth and healthy development can be intuitively reflected in parameters such as weight, organism height, tube circumference, and feed-to-gain ratio (Chen H et al., 2021). Research reported that dietary supplementation of trace metal elements can enhance growth performance and intestinal development (Zhou et al., 2022). As shown in Table 6, and Table 7, dietary addition of RJP-Cu significantly improved growth performance, with the RJP-Cu group exhibiting significantly higher organism weight and tube circumference compared to the control group (*p* < 0.05), indicating RJP-Cu positive effects on lamb growth, development, and health. Evaluating the organism’s antioxidant capacity involves measuring resistance to oxidative stress and its ability to combat free radical damage. Key indicators for this purpose include T-AOC, CAT, GSH-PX, and T-SOD. These enzymes clear free radicals produced during metabolism, maintain the organism oxidation balance, and enhance its antioxidant capacity (Franco et al., 2020). As indicated in Table 8, the levels of T-SOD, T-AOC, GSH-Px, and CAT in the RJP-Cu group were significantly higher than those in the control group (*p* < 0.05). These indicates that RJP-Cu can enhance the activity of antioxidant enzymes, effectively clearing metabolically produced free radicals *in vivo*.

**TABLE 5:** Results of routine blood markers in lambs.

Group/mg/kg	WBC/10^9^/L	LYM/10^9^/L	MON/10^9^/L	NEU/10^9^/L	RBC/10^12^/L	HGB/g/L	PLT/10^9^/L
Control	18.51 ± 5.55^ab^	13.33 ± 4.09^a^	0.09 ± 0.03^ab^	5.72 ± 2.53^ab^	4.34 ± 1.08^a^	9.78 ± 1.37^a^	406.50 ± 57.27^a^
CuSO_4_	15.42 ± 3.54^ab^	10.44 ± 3.62^a^	0.07 ± 0.02^b^	4.06 ± 1.14^b^	5.28 ± 1.82^a^	11.13 ± 1.02^a^	466.67 ± 185.36^a^
RJP-Cu-H	19.98 ± 9.54^ab^	16.12 ± 7.94^a^	0.10 ± 0.05^ab^	3.76 ± 2.58^b^	5.27 ± 2.81^a^	10.40 ± 1.75^a^	486.33 ± 37.04^a^
RJP-Cu-M	13.95 ± 4.30^b^	10.65 ± 3.16^a^	0.07 ± 0.02^b^	3.23 ± 2.13^b^	6.38 ± 2.20^a^	11.15 ± 1.39^a^	479.66 ± 53.53^a^
RJP-Cu-L	23.65 ± 7.25^a^	15.99 ± 4.63^a^	0.12 ± 0.04^a^	7.54 ± 3.30^a^	6.51 ± 2.10^a^	11.37 ± 1.37^a^	454.47 ± 61.36^a^

Note: a, b letters indicate a significant difference (*p* < 0.05).

**TABLE 6:** Effects of RJP-Cu on daily weight gain and feed intake in lambs.

Group/Indicator	IBM/kg	FBW/kg	ADG (g/d)	F/G	ADFI/g
Control	6.32 ± 0.12^a^	9.90 ± 0.25^d^	85.32 ± 4.10^d^	3.19 ± 0.13^a^	271.90 ± 7.67^d^
CuSO_4_	6.27 ± 0.12^a^	11.88 ± 0.42^b^	132.94 ± 8.44^b^	2.25 ± 0.09^bc^	299.13 ± 10.09^ab^
RJP-Cu-H	6.28 ± 0.10^a^	12.35 ± 0.55^a^	144.44 ± 14.88^a^	2.12 ± 0.16^d^	304.29 ± 9.37^a^
RJP-Cu-M	6.32 ± 0.08^a^	11.62 ± 0.37^b^	125.40 ± 9.36^b^	2.35 ± 0.16^c^	293.17 ± 3.78^b^
RJP-Cu-L	6.35 ± 0.10^a^	10.78 ± 0.18^c^	105.56 ± 5.36^c^	2.67 ± 0.11^b^	281.75 ± 3.46^c^

Note: a, b, c, d letters indicate a significant difference (*p* < 0.05).

**TABLE 7:** Effect of RJP-Cu on lamb organism size indicators.

Indicator/cm	Control	CuSO_4_	RJP-Cu-H	RJP-Cu-M	RJP-Cu-L
Initial body height	41.33 ± 0.82^a^	42.17 ± 0.75^a^	42.33 ± 0.52^a^	42.17 ± 0.75^a^	41.83 ± 1.17^a^
Under height	53.67 ± 1.03^d^	64.83 ± 1.33^a^	63.50 ± 1.38^a^	61.17 ± 0.75^b^	57.33 ± 1.97^c^
Initial body diagonal length	41.67 ± 1.03^a^	41.83 ± 1.17^a^	42.17 ± 0.75^a^	42.33 ± 0.82^a^	41.67 ± 1.03^a^
Unbody oblique length	56.50 ± 1.05^d^	67.33 ± 1.21^a^	67.00 ± 1.26^a^	64.33 ± 0.82^b^	60.67 ± 1.97^c^
Initial bust circumference	46.67 ± 0.52^a^	46.67 ± 0.52^a^	47.00 ± 0.63^a^	47.00 ± 0.63^a^	46.83 ± 0.75^a^
Low chest circumference	60.83 ± 1.17^d^	71.00 ± 0.89^a^	71.33 ± 1.21^a^	67.67 ± 1.03^b^	64.17 ± 1.17^c^
Initial pipe circumference	7.92 ± 0.20^a^	7.83 ± 0.26^a^	7.83 ± 0.26^a^	8.08 ± 0.20^a^	8.00 ± 0.00^a^
Open girth	8.83 ± 0.26^c^	10.42 ± 0.49^a^	10.75 ± 0.42^a^	9.67 ± 0.41^b^	9.00 ± 0.32^c^

Note: a, b, c, d letters indicate a significant difference (*p* < 0.05).

**TABLE 8:** Effect of RJP-Cu on serum oxidative stress markers in lambs.

Items	Control	CuSO_4_	RJP-Cu-H	RJP-Cu-M	RJP-Cu-L
T-AOC/(U/mL)	0.68 ± 0.05^b^	0.77 ± 0.02^a^	0.81 ± 0.04^a^	0.76 ± 0.03^a^	0.75 ± 0.01^a^
CAT(U/mL)	2.41 ± 0.93^b^	4.86 ± 2.22^ab^	5.60 ± 2.56^a^	6.97 ± 0.91^a^	6.14 ± 2.04^a^
GSH-Px (U/mL)	656.25 ± 88.42^a^	523.13 ± 89.66^ab^	343.13 ± 181.70^b^	384.38 ± 154.96^b^	541.88 ± 98.05^ab^
T-SOD (U/mL)	17.39 ± 2.25^b^	27.56 ± 4.07^a^	25.68 ± 6.70^a^	26.40 ± 3.64^a^	29.49 ± 3.65^a^
MDA (nmol/mL)	0.21 ± 0.05^b^	0.31 ± 0.11^ab^	0.37 ± 0.17^ab^	0.41 ± 0.12^a^	0.26 ± 0.05^ab^

Note: a, b letters indicate a significant difference (*p* < 0.05).

Immunoglobulins are proteins produced by the immune system, playing vital roles in immune functions. IgA serves as a primary defense antibody on the mucosal surface of the intestines, safeguarding the delicate intestinal lining through the neutralization of potential pathogens or harmful bacteria (Bohländer et al., 2021). lgG is the main immunoglobulin in serum and body fluids, which can enhance immune cells to phagocytose pathogenic microorganisms and fight infection (Gkoliou G et al., 2023). As shown in Figure 7, the RJP-Cu-H led to significantly higher IgA and IgG secretion compared to the control group (*p* < 0.05). These data suggest that RJP-Cu-H could regulate the intestinal immune response by stimulating IgA and IgG secretions. The mucosal cytokine axis strictly governs the intestinal immune system and its defense against invading pathogens (Silva DMD et al., 2021). IL-2 enhances T-lymphocyte activity, improving body immunity (Shapiro MR et al., 2023). IL-4 activate the humoral immune response, induce the expression of immunoglobulin IgG and reduces inflammation (Gärtner et al., 2023). TNF-α promotes the body’s ability to resist infection by pathogenic microorganisms and promoting mucosal immune protection (El-Gindy YM et al., 2023). As shown in Figure 8, RJP-Cu-H group significantly increased the serum secretion of IL-2, IL-4, and TNF-α compared to the control group (*p* < 0.05). These results demonstrate that RJP-Cu effectively enhancing the intestinal immunity through promotes antibody and cytokines secretion.

The intestinal flora plays a vital role in protecting the physiological and immune functions of the gut, and maintaining gut health, and enhancing the growth performance of lamb (Li D et al., 2023). Studies have shown that lambs were feeding with herbal polysaccharide can increase the abundance of flora, promote intestinal immunity and intestinal barrier function (Chen Q et al., 2021). Mice feeding with polysaccharide-iron chelates can increase the abundance of bacteria, promote intestinal immunity and intestinal barrier function (Yuan S et al., 2022). Thus, we were interested whether RJP-Cu could influence the intestinal micro-biome. Therefore, 16S rRNA sequencing technology was employed to study the effects of RJP-Cu on the intestinal microbiome in lamb. Results showed that the coverage for each group is greater than 0.995 and there is no significant difference among each groups (*p* > 0.05), indicating that the sequencing results for each group are complete and reliable (Figure 9B). In β-diversity analysis, the Venn diagram was used to calculate the percentage of common and unique microflora different among groups, the PCoA and NMDS can indicate the magnitude of microflora different among groups, and UPGMA cluster analysis can represent the clustering of similar microflora among groups (Andrei et al., 2021). As shown in Figure 9A, the proportion of unique species in RJP-Cu, Control and CuSO_4_ groups was 2349 species, 1793 species and 1845 species respectively. The difference between RJP-Cu, Control and CuSO_4_ groups was large, and the clustering difference was obvious (Figures 9C, D). The above results indicate that the RJP-Cu group, Control group and CuSO_4_ group had differences in intestinal flora diversity, and RJP-Cu could enhance the intestinal microbial community diversity. In Alpha diversity analysis Chao1, Pielou_e, Observed_species, and Faith_pd are used to represent the total number of species in sequencing samples, Shannon and Simpson indices represent the diversity (richness) of species in the sample (Zhang L et al., 2023). As shown in Figure 9B, Chao1, Pielou_e, Observed_species, Faith_pd, Simpson index and Shannon index in RJP-Cu group are significantly higher than those in the control group (*p* < 0.05). The above results indicate that RJP-Cu significantly increased the diversity and richness of the intestinal flora in lambs. To further investigate the effect of RJP-Cu on the intestinal flora composition of lambs, the intestinal flora composition and relative abundance were analyzed at the phyla and genus levels.

*Bacteroidetes* and *Spirochaetes* play an important role in the fermentation of complex polysaccharide substances, contributing to the maintenance of microbial balance in the gut and the production of SCFAs to support the health and function of colon cells (Ye et al., 2022; Pan et al., 2023). As shown in Figure 10A, at the phylum level, the abundances of *Bacteroidetes* and *Spirochaetes* in RJP-Cu treatment group were higher than that in CuSO_4_ and control group, and the abundance of *Actinobacteria* in RJP-Cu treatment group were remarkably lower than that in control group. These data suggested that RJP-Cu effectively enhances the presence and quantity of beneficial bacteria, reduce the presence and quantity of harmful bacteria *Actinobacteria*, thus positively influencing intestinal flora health. At the genus level, RJP-Cu treatment group significantly increased the abundance of beneficial bacteria *Prevotella*, *Oscillospira*, *Ruminococcus*, *Coprococcus*, *Akkermansia*, and reduced the abundance of *Clostridium* compared to the control group and the CuSO_4_ group (Figure 10B). *Ruminococcus* are known to be responsible for degrading polysaccharides and fibers (Fu X et al. (2018), *Prevotella* is able to promote intestinal mucosal healing. (Hattori S T, et al., 2020). *Oscillospira* has been proven to produce SCFAs dominated by butyrate (Yang et al., 2021). *Coprococcus* is able to modulate the intestinal immunity (Lu H et al., 2022). *Akkermansia* can inhibit intestinal inflammation and promote intestinal immunity (Liu S et al., 2023). *Clostridium* comprises a broad genus of Gram-positive anaerobic bacteria, and the increase of *Clostridium* in the intestinal of animals has a risk of causing severe intestinal infections (Liao X et al., 2023). Therefore, these data supported that the improvement of the intestinal immunity in lamb might be due to the ratio of beneficial bacteria increases and the percentage of intestinal pathogenic bacteria decreases at the same time.

SCFAs were major produced by intestinal microbial fermentation and are essential for maintaining intestinal health and preventing the colonization of some intestinal pathogens. (Cho KM et al., 2021). Valeric acid can improve the morphology of the small intestinal mucosa and reduce intestinal inflammation, the isovaleric acid and isobutyric acid regulate the production of immunoglobulin IgA and a variety of cytokines, and regulate the intestinal immune function (Zhang D et al., 2023). Propionic acid helps maintain the health of the intestinal mucosa and has a active impact on the immune system (Schulthess J et al., 2019). As shown in Table 9 the levels of isovaleric acid, valeric acid, propionic acid, and isobutyric acid in the RJP-Cu group were significantly higher than those in the control group (*p* < 0.05), indicating that RJP-Cu enhances intestinal immune function and intestinal barrier protective function by increasing the concentration of SCFAs in the intestine.

**TABLE 9:** Results of different SCFAs content in each group.

Group	Control	RJP-Cu	CuSO_4_
Acetic acid (μg/g)	1090.46 ± 144.96^a^	1260.95 ± 138.50^a^	1269.04 ± 107.99^a^
Butyric acid (μg/g)	45.63 ± 19.55^a^	68.95 ± 22.93^a^	66.12 ± 11.03^a^
Isovaleric acid (μg/g)	15.10 ± 3.19^b^	22.65 ± 1.82^a^	16.88 ± 2.96^b^
Valeric acid (μg/g)	15.67 ± 4.12^b^	29.44 ± 4.12^a^	23.95 ± 5.49^ab^
Propionic acid (μg/g)	148.77 ± 15.41^b^	269.18 ± 46.38^a^	250.99 ± 20.33^a^
Isobutyric acid (μg/g)	29.10 ± 6.62^b^	37.85 ± 5.13^a^	32.86 ± 3.33^ab^

Note: a, b letters indicate a significant difference (*p* < 0.05).

## 5 Conclusion

In conclusion, RJP-Cu with the highest Cu^2+^ content can be prepared under the conditions of 0.5 g of sodium citrate, a reaction temperature of 50°C, and a pH value of 8. Dietary RJP-Cu can improve growth performance, antioxidant capacity, serum immune function, intestinal flora diversity, intestinal beneficial bacteria quantity and SCFAs secretion of lambs. The above results indicate that RJP-Cu can be used as an effective copper supplement feed additive to promote growth performance and enhance intestinal immunity in lambs.

## Data Availability

The original contributions presented in the study are included in the article/Supplementary material, further inquiries can be directed to the corresponding author.
